# P02.122. Mindfulness meditation for pediatric chronic pain: effects and precautions

**DOI:** 10.1186/1472-6882-12-S1-P178

**Published:** 2012-06-12

**Authors:** B Golianu, L Waelde

**Affiliations:** 1 Stanford University Medical Center, San Carlos, USA; 2 Palo Alto University, Palo Alto, USA

## Purpose

Although there is a substantial literature about the effectiveness of psychological therapies such as relaxation for pediatric chronic pain and about mindfulness meditation (MM) for health and mental health conditions, there has been little systematic attention to the use of MM for pediatric chronic pain. This presentation will address lessons learned from our ongoing clinical trial of MM in a pediatric chronic pain service at a university clinic.

## Methods

We present case material from our ongoing pilot clinical trial of a manualized mindfulness meditation intervention, called Inner Resources for Coping with Chronic Pain (Waelde, 2011). Participants are 30 patients diagnosed with chronic pain and aged 11 – 17 receiving a 6-week, group-based meditation intervention that includes daily home practice of the techniques. Case material from two participants will be presented to illustrate effects and precautions.

## Results

Our case material indicates that patients are able to learn and practice the MM techniques, with good adherence to the home practice. A male patient with multiple pain complaints was able to learn and practice the meditation techniques and use them to cope with stressors associated with his medical conditions. A 16 year old female patient with longstanding participation in pain management that primarily involved distraction found the concurrent use of mindfulness and distraction to be confusing and ineffective.

## Conclusion

MM shows promise as an intervention for helping children and adolescents to deal with chronic pain. The children and adolescents in our clinical trial have evidenced the ability to engage with the material and adhere to regular home practice of the techniques and application of them to presenting problems. However, a note of caution is raised by the possible incompatibility of mindfulness with concurrent therapies emphasizing the use of distraction for dealing with chronic pain.

